# Identification of Road-Surface Type Using Deep Neural Networks for Friction Coefficient Estimation

**DOI:** 10.3390/s20030612

**Published:** 2020-01-22

**Authors:** Eldar Šabanovič, Vidas Žuraulis, Olegas Prentkovskis, Viktor Skrickij

**Affiliations:** 1 Transport and Logistics Competence Centre; Vilnius Gediminas Technical University, Saulėtekio al. 11, LT-10223 Vilnius, Lithuania; eldar.sabanovic@vgtu.lt (E.Š.); vidas.zuraulis@vgtu.lt (V.Ž.); viktor.skrickij@vgtu.lt (V.S.); 2 Department of Mobile Machinery and Railway Transport, Vilnius Gediminas Technical University, Plytinės g. 27, LT-10105 Vilnius, Lithuania

**Keywords:** video image sensor, vehicle perception system, road type identification, artificial intelligence, vehicle dynamics

## Abstract

Nowadays, vehicles have advanced driver-assistance systems which help to improve vehicle safety and save the lives of drivers, passengers and pedestrians. Identification of the road-surface type and condition in real time using a video image sensor, can increase the effectiveness of such systems significantly, especially when adapting it for braking and stability-related solutions. This paper contributes to the development of the new efficient engineering solution aimed at improving vehicle dynamics control via the anti-lock braking system (ABS) by estimating friction coefficient using video data. The experimental research on three different road surface types in dry and wet conditions has been carried out and braking performance was established with a car mathematical model (MM). Testing of a deep neural networks (DNN)-based road-surface and conditions classification algorithm revealed that this is the most promising approach for this task. The research has shown that the proposed solution increases the performance of ABS with a rule-based control strategy.

## 1. Introduction

Currently, the automotive industry is facing the challenge of automated driving. Achievements in fields of vehicle dynamics, control engineering, and artificial intelligence enable implementation of this technology. However, automated driving requires combined sensing solutions and hardware for perception. Vehicle systems such as brakes, steering, and active suspension, can be improved significantly and implemented in the automated vehicle without additional cost only by using perception.

Road friction estimation is a useful tool for various aspects in driving safety alerting a driver about the road-surface conditions, modifying vehicle active safety systems thresholds or reporting information to a vehicle or road infrastructure network. The applications of such technologies have been already introduced for patenting and near future implementation in new vehicle production [1].

Nowadays, active systems in the vehicle are actuated after estimating vehicle dynamic parameter response generated by the impact and, as a result, actuators have only several milliseconds to tune their characteristics. Actuators used in such systems are complex, expensive, and consume a lot of energy. If the vehicle receives the data about the driving conditions before reaching the place where the control should be applied, it has more time to select optimal tuning parameters. As a result, the comfort level for passengers, and their safety may be increased, even using slower, but cheaper actuators [2].

Road pavement type and its condition identification is a vital task for brake system performance. There are several groups of sensors available for this task. The first group of sensors that can be used is inroad. Such sensors are placed in the road surface and can measure surface temperature, evaluate coating, and other parameters. Data received from inroad sensors can be sent to the vehicle using vehicle-to-infrastructure (V2I) and vehicle-to-roadside (V2R) communication [3,4]. Tanizaki et al. [5] used a vibration sensor placed under the road surface for tyre type recognition. The authors managed to discern winter from summer tyres. It is possible to implement the same technology to identify pavement coating. Stationary video cameras with different filters can be used for surface evaluation. Colace et al. [6] introduced and tested an original approach for the optical assessment of road conditions due to various atmospheric perturbations. The sensing system was based on measuring diffused and reflected light under near-infrared illumination, and extracting the polarisation contrast after reflection.

The second group of sensors is in-vehicle. Wang et al. [7] proposed two different approaches for road pavement type and its coating identification using two approaches: (i) effect-based—identifies road friction conditions through estimating dynamic parameter response of the vehicle; (ii) cause-based—detects causes, before they affect road friction, using various sensors. The main advantage of the cause-based approach is road friction conditions identification before reaching the measured surface point. A conventional ABS is an effect-based system because it uses sensor information about vehicle velocity, wheel angular velocity, acceleration, and wheel slip, and its performance may be improved combining effect-based and cause-based approaches.

Bhandari et al. [8] used the effect-based approach for surface prediction. The authors compared the measured coefficient of friction to the calculated value. The authors used six main types of road surfaces: dry/wet asphalt, dry/wet cobblestone, snow, and ice in their investigation. The only drawback may be the unnecessary loss of braking while checking for the surface change when the surface does not change. Alonso et al. [9] and Kalliris et al. [10] proposed the road classification system based on real-time acoustic analysis of tyre/road noise. This system can identify dry and wet asphalt surfaces with good accuracy. Theoretically, it is possible to identify if the surface is icy and snowy. Ngwangwa and Heyns [11] used acceleration sensors and an artificial neural network for estimating the condition of a road surface by approximation of its profiles and their roughness classes utilising displacement spectral densities. Taniguchi et al. [12] proposed the use of an ultrasonic distance sensor for monitoring road-surface conditions. The authors used a low-cost ultrasonic sensor to measure the road-surface roughness. Such a system may be useful in the prevention of accidents if the information on bad road-surface conditions, such as break, potholes, obstacles, bumps, is obtained in advance. The main advantage of such a system is that a road surface can be measured in the front of the moving vehicle before the front wheel contact with an obstacle. Niskanen and Tuononen [13] proposed the friction identification by estimating a three-axis accelerometer mounted inside the tyre. While such a method can be applied to detect friction potential indicators, different levels of pavement roughness still cause undesirable vibration and a negative influence on results. As an alternative to the accelerometer, the strain gauges have been mounted in the inner liner surface of the tyre to characterise the grip [14]. The experimental research showed strong straining parameters relation with tyre lateral force but for limited grip. While widely used vehicle active safety systems such as ABS, a traction control system (TCS), and an electronic stability program (ESP), have already used road friction estimation during initial cycles of its performance, the rapid development of ADAS technologies requires friction information in advance [15]. To maintain sufficient safety and comfort, the grip level must be estimated earlier, during free-rolling. However, slip-based approaches are insufficient for high excitation levels including road roughness in wet conditions. After experimental tests of vehicle state estimation including tire-road friction coefficient, significant inaccuracies of estimation during sharp cornering were detected [16].

Sensors in the automated vehicle are used for perception, and most of them are cause-based: an ultrasonic sensor; video, thermal and stereo cameras; radars; laser-based radar (LIDAR), a global positioning system (GPS), etc. Video cameras are usually used for determining path [17] and obstacles [18], line detection and road edge recognition [19,20]. Also, there are developments where image analysis methods are used for road distress, cracks and other road damage [21,22,23]. Most image processing can be done using traditional image-processing methods such as histograms, thresholding and other [24], but a currently emerging trend is the use of deep neural network (DNN)-based methods for feature extraction, image matching and decision making [25]. Similar research [26] included recognition of road type and quality but not conditions. Researchers used their small dataset of 512 images per class for road type classification and 221 for road quality classification, and the dataset was collected from Google Street View. Previously, visual recognition has been used for detection of bad road visibility conditions, weather and lighting conditions on roads [27,28]. A SqueezeNet model, as one of the deep learning models, was established as the most accurate model for road-surface condition estimation comparing with CNN and feature-based models [29]. By decreasing the number of input channels it was also the fastest, but more sensitive to training data compared with CNN architecture. A not significantly large number of 100 sets of high-quality road images were used to train the model for estimation of pavement friction level [30]. However, a DNN based on domain knowledge analysis method performed with high enough accuracy of 90.67%, but only by using additional double image distribution. There are datasets available online, for example, Karlsruhe Institute of Technology and Toyota Technological Institute (KITTI) dataset [31], Udacity dataset [32], Oxford Robot Car team dataset [33], the Malaga Dataset [34] and can be used in the development of new processing algorithms, also simulated/synthetic data can be used from self-driving car simulators such as LG Silicon Valley Lab (LGSVL) simulator [35]. However, none of the above-reviewed datasets and simulators fully matched requirements for road pavement type and condition dataset samples. Therefore, the dataset presented in [2] was used during research.

To check the efficiency of the idea, that vehicle brake system with a preview option is effective, the mathematical model (MM) is needed. Wang et al. [7] and Bhandari et al. [8] used Burckhardt tyre model [36], which was developed using the fitting of experimental data collected from a large number of roads. The Pacejka Magic formula can also be used instead of the Burckhardt model. In both cases, numerical values of coefficients are needed. These coefficients can be identified solving optimisation task using experimental data [37]. Cabrera et al. [38] analysed the friction-slip curve as a function of speed using the Magic formula. After a tyre model is developed, the ABS algorithm is needed. The algorithm presented in [39] was used as a reference. The extensions of this algorithm in different vehicle dynamics cases are used by other researchers as well [40].

The next step is the development of the MM of the whole vehicle. Such a model is needed for comparison of a conventional ABS and a system with a preview option. Most commonly used models with 2, 4, 14 and 38 degrees of freedom (DOF). The simplest one is the quarter-car model [41] which has 2 DOF. Displacements of the unsprung mass and a quarter of the car body are included. The 4 DOF model is a half-car model, where the car body has 2 DOFs (displacement in a vertical direction and one rotation), and one DOFs for each wheel vertical displacement. The 14 DOF model consists of 6 DOFs of the vehicle (longitudinal, lateral, vertical motions, also pitch, roll, yaw rotations), 4 DOFs vertical motions of unsprung masses, and the remaining 4 DOFs are the rotations of wheels [42]. The 38 DOFs consist of 6 DOFs of vehicle, and 8 DOF for each wheel [43]. In this research vertical and longitudinal vehicle dynamics have been taken into account.

The main task of this investigation was to improve vehicle ABS performance using additional data from the video image sensor. The system of real-time road-surface classification using visual data and DNN was developed and applied for the vehicle brake system. The investigation of created system efficiency was provided using MM of a vehicle, tyres and ABS, MMs were validated using experimental data.

## 2. Materials and Methods

Blaupunkt BP 3.0 FHD GPS car camera, mounted on the front window of the car, was used for capturing videos for dataset creation. Video files were recorded using 1920 × 1080 px resolution 30 frames per second (FPS) settings. This camera has a 140° diagonal field of view wide-angle lens, OmniVision OV2710 1/2.7-inch 2 MP complementary metal-oxide-semiconductor (CMOS) video image sensor [44]. This sensor has 3 μm pixels, with low-light sensitivity of 3700 mV/lux-s, the signal-to-noise ratio of 40 dB and a peak dynamic range of 69 dB [45]. Car camera records video using h.264 codec. As the characteristics suggest, the camera should provide good quality in low light situations, but unfortunately, very lossy compression leads to high detail loss in bright and dark image areas. This camera has no selectable compression quality level nor bitrate, and uncompressed video cannot be recorded. While examining raw image frames, blurred frames due to vibrations, and low-detail frames due to dark conditions were found, some of the fragmented blurs appeared due to compression using h.264 codec.

Data were collected during different seasons to capture different kinds of weather conditions and road surfaces. The processing of raw data to prepare the dataset consisted of a few steps. Firstly, the videos were split into images and these images were sorted into six categories manually. Secondly, all excessively blurred images and images that were too dark were removed. This is due to the fact, that camera requires an external light source that is stronger than car headlights and light is not coming from the camera side or shining directly into the camera. Finally, a created dataset was split into training, validation, and test parts. The validation and training samples were selected by cutting random sequences of images from the full dataset, and the reminding was assigned to the training part. The prepared dataset consisted of 12,440 images separated into training (10,040), validation (1200) and testing (1200) parts, that were in separate folders inside which image samples sorted into folders by class. Created training and validation parts were used for training and measurement of developed algorithm performance, and testing parts were used for its performance evaluation. Such a distribution of data in training and validation parts led to better evaluation of the actual performance of the created models. This approach guaranteed that validation and training samples were not too similar to the training samples, but were from a similar situation, so samples training, validation and testing parts belonged to the same distribution.

### 2.1. Creating Deep Neural Network (DNN)-Based Classification Algorithm

**Figure 1:**
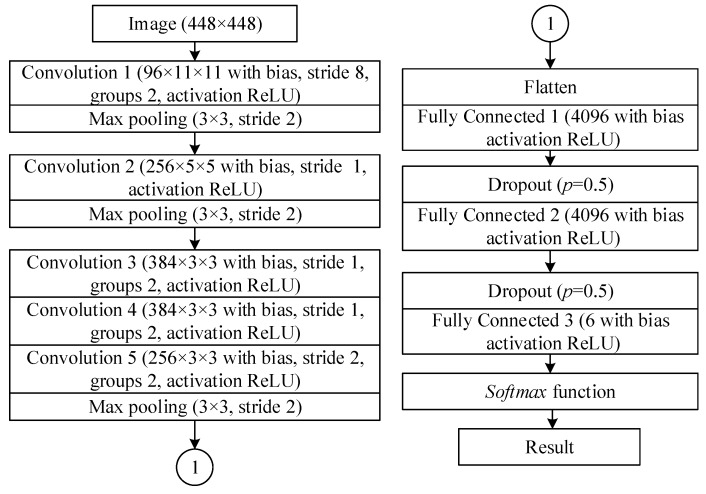
Deep neural network (DNN) model for road type and conditions classification.

An emerging artificial intelligence method of deep learning has been used for classification of six road-surface types and condition combinations: gravel wet, gravel dry, cobblestone wet, cobblestone dry, asphalt wet, asphalt dry; and is presented in this subsection. The convolutional neural network (CNN) model Alexnet, with modifications to process a bigger image, was used for image classification. The structure of the DNN model used is presented in Figure 1. The model was designed and trained using the Nvidia Deep Learning Graphics Processing Unit (GPU) Training System (DIGITS) [46]. This web-based user interface and underlying middle layer allow fast dataset preparation with training, validation, testing parts, prototyping of model using Caffe [47] or TensorFlow [48]. The developed DNN was trained using workstation with an Nvidia Geforce 2080 Ti GPU. The final model was converted using an Nvidia TensorRT [49] and tested on an Nvidia Jetson TX2 embedded system [50]. This embedded system consumes only up to 15 W of power and provides up to 1 TFLOP of DNN calculations. Also, it has hardware pipelines for camera video stream decoding and video file stream decoding and encoding that can be deployed in-vehicle for real-time image analysis.

The developed DNN model is made of five convolutional layers and three fully connected layers, all with rectified linear units as the activation function. To reduce classifier dependence on separate pixels, dropout layers were used. The main differences of developed DNN model compared to Alexnet is increased input image size of the first convolutional layer from 227 × 227 px to 448 × 448 px and stride increase from 4 to 8 of the same layer. A bigger input image permits better performance as provided by smaller details, and also local response normalization was removed as it proved to lead to no performance improvement for the task.

Convolution operations are implemented in a similar way to human vision feature extraction and processing capabilities in computer vision. This operation enables the learning of spatial feature representations for specific image processing tasks. During convolution operation, kernels are being shifted over an image and matrix multiplication operation between kernel and image part values that kernel is currently on, afterwards multiplication results are summed and processed by a selected activation function.

Max pooling layers allows for maximal value selection in local image field to improve shift and rotation invariance. The dropout operation between fully connected layers is used for improving stability of features, lowering dependency on single values by randomly zeroing half of the outputs of previous layers while training the network, and using a multiplication coefficient to achieve same signal level between layers during inference.

To train this DNN, a stochastic gradient descent algorithm was used with a base learning rate of 0.01, that was reduced by a factor of 0.3 every 4 from a total of 20 epochs. After training, the best performance snapshot after 28th epoch was selected because of the lowest loss value on validation data.

The input image was scaled and the pixel mean normalised before processing with the DNN-based algorithm. DNN input was a 3 channel image of 448 × 448 px. During training and validation process, the input image was cropped from training sample of 512 × 512 px size at random coordinates; these created more unique samples and reduced the possibility of overfitting. The softmax function was used for class selection. It provides normalised probabilities of a road in input image belonging to all classes and selects the class with the highest probability. Probability values can be used for further determination of reliability of classification results. Mean filtering may be used for single false classification removal.

### 2.2. System Implementation

**Figure 2:**
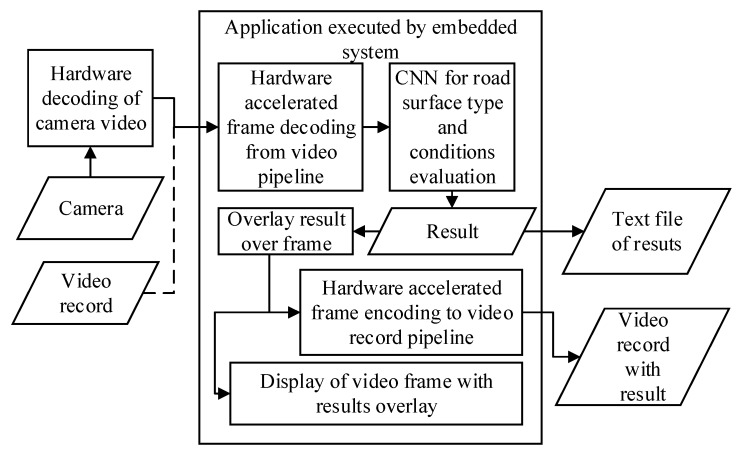
Blocks schematic of system implementation.

An application that reads input images, analyses them using the CNN-based algorithm, and saves the results are presented in this subsection. The application that implements the CNN based evaluation algorithm for real-time camera image or video record image processing, was written in C++. In Figure 2, a block schematic of this application is presented. GStreamer pipelines with support of hardware decoding and encoding were used to reduce CPU load. GStreamer is a library that provides ways for creating video preprocessing, encoding and decoding including support for acceleration using hardware pipelines. These hardware pipelines configured to have direct access to camera and memory, without raised CPU usage. Road images can be streamed from camera or video record file while testing. A video encoding pipeline is used for result presentation on screen and recording to video file. In addition, results can be written to file or streamed over the network to controller. The basics of image recognition implementation were taken from the Nvidia tutorial [51] which shows how to use DIGITS and TensorRT for implementation of CNN models on the Jetson TX2 for real-time inferencing.

The created application supports command line parameters that allow fast inference testing using different DNN models. In addition, it supports processing of recorded video files and video, captured with a camera in real-time for the deployment in-vehicle for real-time road pavement type and condition evaluation. The results are calculated for each provided image at the input, no additional filtering or post-processing was used while testing.

### 2.3. Vehicle Mathematical Model

**Figure 3:**
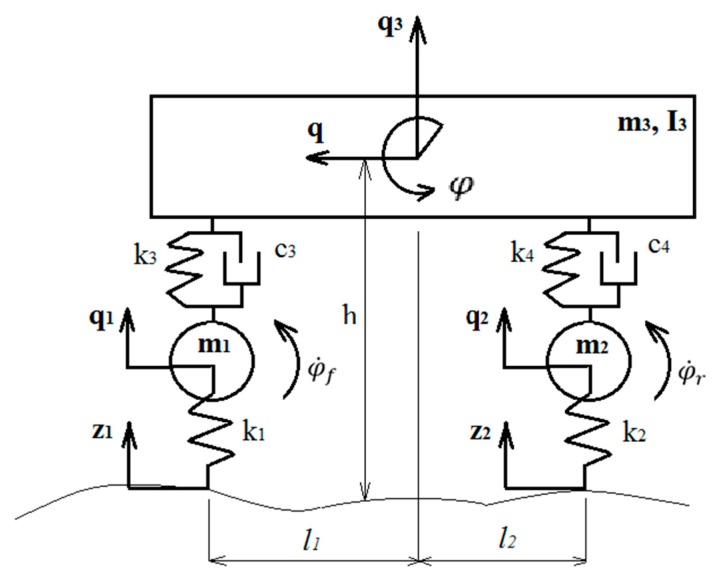
Vehicle dynamic model.

Vehicle MM used for performance evaluation of the developed road-surface type identification system is presented in this subsection. The car dynamic model with 7 (DOF) was developed (Figure 3) during the investigation. For vertical dynamics investigation the 4 DOF car model is used; one DOF for longitudinal dynamics, and the two DOF model for the wheels’ rotation. Toyota Prius was used as a reference vehicle during the simulation. Vehicle parameters used for the MM are presented in Table 1.

The first assumption, vehicle cornering (lateral displacement) was not taken into account in the MM. The second assumption, the longitudinal acceleration of the vehicle, is equal to the longitudinal acceleration of the sprung mass.

Verticals forces $F_{zf}$ and $F_{zr}$ were calculated using equations of motion:

(1) $$m_{1}{\ddot{q}}_{1}\;=\;k_{3}\left(q_{3}\;-\;l_{1}\varphi \;-\;q_{1}\right)\;+\;c_{3}\left({\dot{q}}_{3}\;-\;l_{1}\dot{\varphi }\;-{\dot{\;q}}_{1}\right)\;-\;m_{1}g\;-\;k_{1}\left(q_{1}\;-\;z_{1}\right);$$

(2) $$m_{2}{\ddot{q}}_{2}\;=\;k_{4}\left(q_{3}\;+\;l_{2}\varphi \;-\;q_{2}\right)\;+\;c_{4}\left({\dot{q}}_{3}\;+\;l_{2}\dot{\varphi }\;-\;{\dot{q}}_{2}\right)\;-\;m_{2}g\;-\;k_{2}\left(q_{2}\;-\;z_{2}\right);$$

(3) $$\begin{array}{c} m_{3}{\ddot{q}}_{3}=\;-k_{3}\left(q_{3}\;-\;l_{1}\varphi \;-\;q_{1}\right)\;-\;c_{3}\left({\dot{q}}_{3}\;-\;l_{1}\dot{\varphi }\;-\;{\dot{q}}_{1}\right)\;-\;k_{4}\left(q_{3}\;+\;l_{2}\varphi \;-\;q_{2}\right)\\ -\;c_{4}\left({\dot{q}}_{3}\;+\;l_{2}\dot{\varphi }\;-{\dot{q}}_{2}\right)\;-\;m_{3}g; \end{array}$$

(4) $$\begin{array}{c} I_{3}\ddot{\varphi }=k_{3}l_{1}\left(q_{3}\;-\;l_{1}\varphi \;-\;q_{1}\right)\;-\;c_{3}l_{1}\left({\dot{q}}_{3}\;-\;l_{1}\dot{\varphi }\;-\;{\dot{q}}_{1}\right)\;-\;k_{4}l_{2}\left(q_{3}\;+\;l_{2}\varphi \;-\;q_{2}\right)\\ -c_{4}l_{2}\left({\dot{q}}_{3}\;+\;l_{2}\dot{\varphi }\;-{\dot{q}}_{2}\right)\;-\;m_{3}g-F_{f}l_{1}+F_{r}l_{2}; \end{array}$$

where $m_{1}$—front unsprung mass; $m_{2}$—rear unsprung mass; $m_{3}$—sprung mass of a vehicle; $I_{3}$—moment of inertia of the vehicle; g—gravity; $l_{1}$—distance from front wheel to the centre of gravity of the vehicle body; $l_{2}$—distance from the rear wheel to the centre of gravity of the vehicle body; $k_{1,2}$—front and rear tyre stiffness; $k_{3,4}$—stiffness of the front and rear suspension; $c_{3,4}$—damping of the front and rear suspension; $F_{f}\;=\;-\frac{m_{3}\ddot{q}h}{l_{1}\;+\;l_{2}}$ and $F_{r}\;=\;\frac{m_{3}\ddot{q}h}{l_{1}\;+\;l_{2}}$—forces that affect front and rear axles during the deceleration.

Vertical forces acting front and rear axles:

(5) $$F_{zf}\;=\;k_{1}\left(q_{1}\;-\;z_{1}\right)\;and\;F_{zr}\;=\;k_{2}\left(q_{2}\;-\;z_{2}\right)$$

Vehicle longitudinal dynamic equation of motion during the braking:

(6) $$m_{tot}\ddot{q}\;=\;-F_{tot};$$

where $m_{tot}$—is total vehicle mass; $\ddot{q}$—longitudinal vehicle acceleration; $F_{tot}$—total longitudinal tyre friction force:

(7) $$F_{tot}\;=\;\mu_{f}F_{zf}\;+\;\mu_{r}F_{zr}$$

Wheel rotational dynamic equation of motion during the braking:

(8) $$I_{f,r}{\ddot{\varphi }}_{f,r}\;=\;R_{f,r}\mu_{f,r}F_{zf,r}\;-\;T_{bf,r};$$

where $I_{f,r}$—moments of inertia for the front and rear axle wheels; ${\ddot{\varphi }}_{f,r}$—angular accelerations of front and rear axle wheels; $T_{bf,r}$—braking torques on the front and rear axles, for the vehicle under investigation $\frac{T_{bf}}{T_{br}}\;=\;1.5$ (value defined during experiment); $R_{f,r}$—effective radii of front and rear axle wheels.

The wheel slip is defined as:

(9) $$\lambda_{f,r}\;=\;\frac{\dot{q}\;-\;{\dot{\varphi }}_{f,r}R_{f,r}}{\dot{q}}$$

where $\dot{q}$—vehicle longitudinal velocity; ${\dot{\varphi }}_{f,r}$—angular velocities of the wheels on the front and rear axles.

Wheel slip $\lambda_{f,r}$ is required for the evaluation of the friction coefficients. $\mu_{f,r}$. In this paper Magic Formula tyre model proposed by Pacejka was used [37]:

(10) $$\mu \;=\;D\cdot sin\left(C\cdot arctan\left(B\left(\lambda \;+\;S_{h}\right)\right)\;-\;E\left(B\left(\lambda \;+\;S_{h}\right)\right)\;-\;\arctan\left(B\left(\lambda \;+\;S_{h}\right)\right)\right)\;+\;S_{v}$$

where $D,\;C,\;B,\;E,\;S_{h},\;S_{v}$—coefficients evaluated from experimental measurements.

The ABS algorithm proposed in [39] was used in this investigation and it and the control strategy are presented in Appendix A.

### 2.4. Experimental Investigation

**Figure 4:**
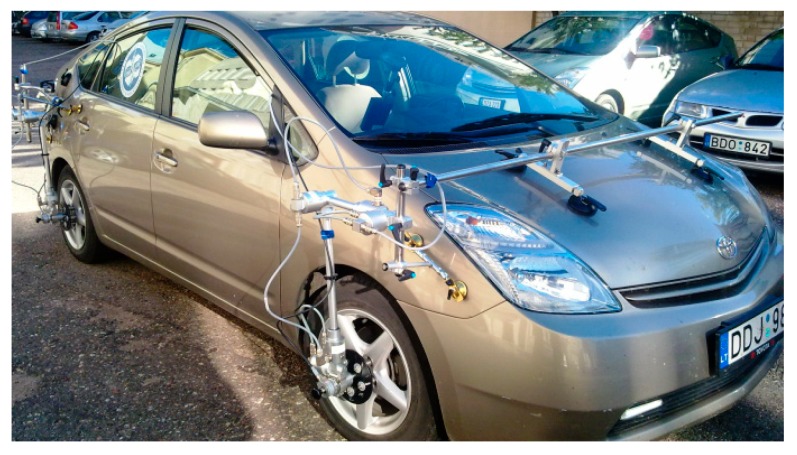
Test vehicle.

To parametrise the vehicle tyre, model an experimental investigation was carried out and is presented in this subsection. A Toyota Prius test vehicle was used during the experiment (Figure 4). It was equipped with Kistler group measurement equipment. Equipment for experimental research included: inertial measurement unit (IMU) for sprung mass acceleration and rate of angular rotations (Corrsys-Datron TANS-3215003M5), a non-contact optical sensor for vehicle speed (Correvit S-350 Aqua), wheel pulse transducer for wheel rotation speed (Corrsys-Datron WPT), laser distance sensor for wheel effective radius (Corrsys-Datron HF-500C). A data acquisition system (Corrsys-Datron DAS-3) was used for data logging with selected 200 Hz frequency.

**Figure 5:**
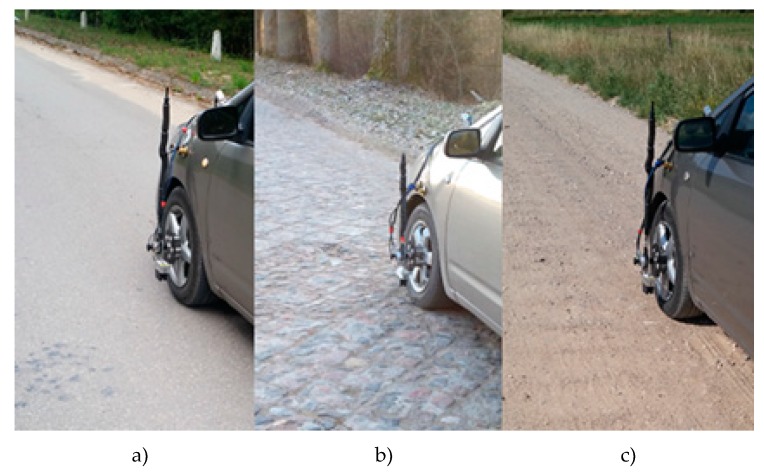
Selected road surfaces for test braking: (a) asphalt, (b) cobblestone, (c) gravel.

Test braking on three different pavements was performed (Figure 5). All the experiments were performed when the road surface was dry and wet.

Each braking test was carried out from the same initial speed keeping constant press force of brake pedal and straight driving trajectory. The data collected during the experiments enabled the determination of longitudinal wheel slip ratio and its representation with vehicle braking efficiency expressed by longitudinal acceleration.

## 3. Results

### 3.1. Evaluation of the DNN-Based Classification Algorithm

The DNN-based road pavement type and condition classification algorithm was tested and the results are presented in this subsection. Results are presented in the form of a confusion matrix with per-class accuracy, precision, recall, F1 score metrics in Table 2. There we can see that the classification accuracy of wet conditions such as asphalt wet, cobblestone wet, and gravel wet were higher compared to dry conditions of same road types. The most errors are made between the same class conditions, especially gravel; dry gravel was confused with wet 47 times out of 200, but less with asphalt or cobblestone. It is hard to discern dry and wet, because a gravel pavement can be of different colour and tone. Dry cobblestone was confused with dry asphalt 15 times, and wet cobblestone with wet asphalt 9 times. This was mainly related to similar colours and the low detail of images because of low light or image motion blur, as during these conditions these road times look very similar. Dry cobblestone dry was confused with wet cobblestone 8 times mostly because of the shadows on the pavement. Results show that DNN-based algorithm provides sufficient performance for the planned use case.

The implemented system provided real-time video processing of 30 FPS, with 20 ms processing time per frame. Average classification accuracy of 6 classes was 88.8% on the validation dataset and 88.3% on the testing dataset. There were no algorithms developed whose performance could be directly compared because usually road type and conditions are classified separately. Therefore, to compare the algorithm’s performance to those developed by other researchers, the road-surface type and conditions classification were evaluated separately in Table 3 and Table 4. Confusion matrix and precision, recall and F1 score metrics are presented. There average road pavement type classification accuracy is 96% and average road condition classification accuracy is 92%. The achieved pavement type classification accuracy is higher compared to accuracy showed by Tumen et al. [26]. In addition, the used dataset is bigger and includes a wider variety of conditions, such as different seasons and time of day, and therefore results may be replicated easier. Achieved average road condition classification accuracy using our vision-based algorithm is less than 5% worse than best results showed by Alonso et al. [9] and Kalliris et al. [10] using acoustic sensors. Also, the achieved condition classification accuracy of 92% was lower compared to Roychowdhury et al. [29] and the visual classification of road conditions accuracy of 97.36%. The authors achieved this result using SqueezeNet to process image patches of 6 color channels in front of the car, while the model that is presented in this paper gets unprocessed input image of 3 colors; it would be a good idea to implement that decision in our future work. Similarly to Alonso et al. [9], our algorithm provides higher accuracy for wet pavement conditions. The algorithm presented in this paper can provide classification results in advance with 20 ms delay, while Alonso et al. [9] reported a response time of 0.2 s and Kalliris et al. [10] did not provide any classification or response times.

The analysed results showed that the classification of forefront road condition is a harder task compared to road-surface type classification. Mostly, wet and dry conditions of same road-surface type class were misclassified, especially gravel. Conditions are misclassified because of non-uniform illumination and shadows. Shadows of viaducts and bridges, as well as dark parts of tunnels, provide major difficulties as well, as they may be mistaken for wet pavement. Therefore, shadow compensation methods may be applied in this sector in future. The road-surface type classification errors are rarer. These errors are caused by the limited dynamic range of a video sensor, slow brightness adaptation, and mostly because of lost details in compression. The worst detail loss happens when there are a lot of details in the image and the compression algorithm cannot preserve all of them.

In summary, results also confirm that the visual spectrum camera has the same drawbacks as human vision. However, this solution can work very well and will not lose attention compared to the human driver. In the case of poor visibility due to mist, heavy rain, hail, direct sunlight the image cannot ensure good identification of the pavement. These conditions can be detected, and then an effect-based sensors should be used instead of the visual spectrum camera.

### 3.2. System Performance Evaluation

**Figure 6:**
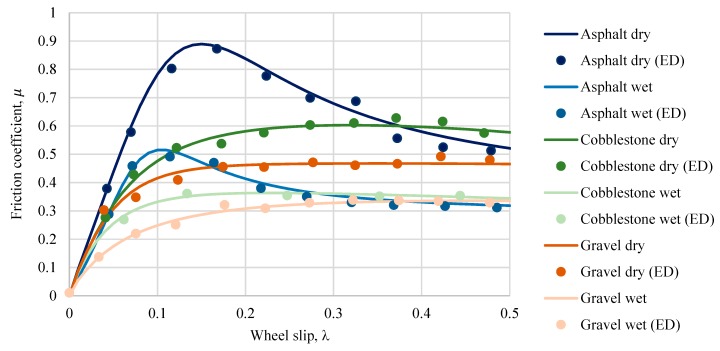
Numerical values of calculated and experimental data (ED) friction coefficients.

During the experiment, the road friction coefficient and longitudinal wheel slip ratio were measured for six different cases under investigation, and the results are presented in this subsection. Results were filtered using the methodology proposed by [37]. Magic formula coefficients (Equation 10) were defined using the non-linear least squares optimisation method. In Figure 6, experimental data is presented with points and marked (ED), and the results achieved using the Magic formula are shown with lines. Experimental data and μ − λ curves achieved using the Magic formula coincide well. However, in [37] the authors yielded even more consistent results, but tests were carried out under laboratory conditions, in our case all the measurements were carried out under real conditions.

### 3.3. Effectiveness of the Proposed Solution

**Figure 7:**
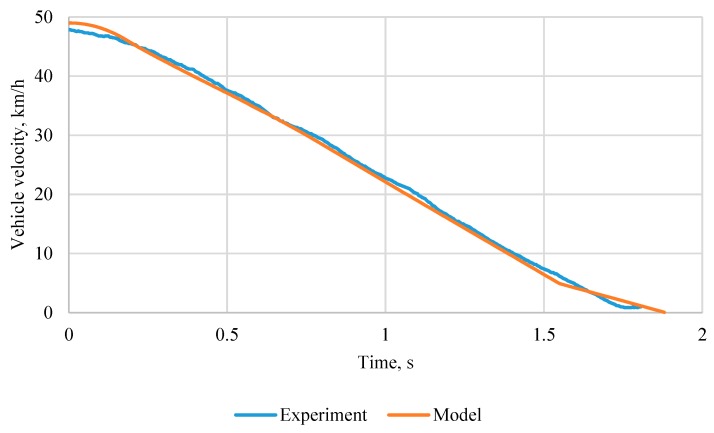
Validation of vehicle MM with anti-lock braking system (ABS).

After the evaluation of coefficients used in the Magic formula for all road surfaces, the MM presented in Section 2.3 was developed and validated using experimental data presented in Figure 7. During the validation numerical values of the main ABS parameters, $a_{min},\;A,\;a_{max},\lambda_{ref}$ were evaluated and braking torque variation intensity was chosen.

As can be seen in Figure 7, the MM reproduces experimental data very well and can be used for further research. Using MM, the braking of the car was simulated on different pavement types, with initial velocity 50 km/h and results are presented in Table 5. It can be seen that in a conventional ABS system with $\lambda_{ref}\;=\;0.15$ performs optimally on dry asphalt and the developed system with preview has no advantage. However, in all the other cases the developed system performs better. The best results were achieved on wet asphalt, where stopping distance decreased by 18%. On wet gravel, the distance decreased 13%, for other cases effect was less than 10%. The car using conventional ABS achieves shorter stopping distance compared to a car without ABS on most road-surface types and conditions, excluding wet gravel. The shorter stopping distance without ABS on wet gravel may be caused by pushing gravel against the wheel.

As shown in Section 3.1. during the classification errors may appear and it may affect the braking distance. In Table 6 stopping distance is presented for cases when classification error appears.

As expected, a significant increase in stopping distance appeared. Only on dry cobblestone and dry/wet gravel did the developed system perform better, for some cases, than conventional ABS (Table 6, first column). To solve this challenge the output data need to be filtered and fused with other in-vehicle sensors. Also, the case of misclassification brings lower probability values for the actual output of the DNN-model, and therefore all results when the DNN-model output probability is lower than the set threshold can be ignored as not trustworthy. In such a case the slip 0.15 should be taken as a reference and the braking distance will be the same as in a conventional system.

## 4. Discussion and Conclusions

A developed road type classification solution based on video data and DNN provides wide application potential in the automotive industry. It uses sensors and hardware that will be already installed in automated vehicles. Information about road type can significantly increase the effectiveness of vehicle steering, braking, acceleration performance as well as stability and safety systems.

A DNN-based algorithm for road pavement type and conditions was developed. This algorithm was tested to achieve 88.8% average classification for 6 combinations of road pavement types and conditions: dry asphalt, wet asphalt, dry cobblestone, wet cobblestone, dry gravel, wet gravel. It achieved even higher accuracy of 96% for 3 road pavement types: asphalt, cobblestone, gravel and 92% for dry and wet classification. This algorithm was implemented and its execution speed tested using Nvidia Jetson TX2, which can be installed in-vehicle to process real-time video. An implemented algorithm was processing one image in 20 milliseconds, which is enough to process up to 50 frames per second, and allows monitoring of each half meter of road pavement at 100 km/h speed. The results can be then used for better control of other systems.

In this article, a case study with braking was under investigation. There are different configurations of ABS available on the market. It has to be admitted that the method proposed in this research will not be suitable for absolutely all braking systems. The numerical value of wheel slip when the friction coefficient is maximal varies. It depends on vertical load, tyre pressure, temperature, surface roughness and other parameters [15,52,53]. Implementation of our developed method in a high-dynamic decoupled electro-hydraulic brake system as presented in Savitski et al. [54] will not be effective. As in such systems, reference wheel slip is calculated defining max longitudinal force, and such factors as load, tyre pressure, the temperature are essentially irrelevant. The main shortcoming of the high-dynamic decoupled electro-hydraulic brake system is its complexity and cost. The main advantage of our solution is that it can be implemented in a simple brake system with a rule-based control algorithm. As shown, the effectiveness of our solution for such a system will be high.

The developed MM was validated and reproduced experimental data well. The stopping distance when braking on different pavements types without ABS, conventional ABS, and developed system were simulated. Results showed that the proposed solution reduced stopping distance for all analysed road types and conditions, excluding dry asphalt, on which the stopping distance was not changed. The best results were achieved on wet asphalt, where stopping distance decreased by 18%.

In future, the new dataset will be created using recorded uncompressed data as will be present during real-time processing in the vehicle. The dataset will include snowy and icy conditions as well as more complicated environments for camera sensing such as sunshine, twilight, fog or mist. The DNN-model will be improved to use fewer resources and perform with higher accuracy and speed. The algorithm safety and reliability will be improved by adding faulty classification detection and filtering. The feature system may use data fusion of the DNN-based algorithm’s result and in-vehicle sensors.

## Figures and Tables

**Table 1:** Parameters for mathematical model (MM).

Parameter	Numerical Value
$\mathrm{Unsprung}\;\mathrm{mass}\;\mathrm{of}\;\mathrm{front}\;\mathrm{wheels},\;m_{1}\;\left[\mathrm{kg}\right]$	60
$\mathrm{Unsprung}\;\mathrm{mass}\;\mathrm{of}\;\mathrm{rear}\;\mathrm{wheels},\;m_{2\;}\left[\mathrm{kg}\right]$	40
$\mathrm{Vehicle}\;\mathrm{body}\;\mathrm{mass},\;m_{3}\;\left[\mathrm{kg}\right]$	1160
$\mathrm{Vehicle}\;\mathrm{body}\;\mathrm{inertia}\;\mathrm{moment},\;I_{3}\;\left[{\mathrm{kgm}}^{2}\right]$	1761.4
$\mathrm{Gravitational}\;\mathrm{acceleration},\;g\;\left[m/s^{2}\right]$	9.81
Stiffness of front wheels, $k_{1}\;\left[N/m\right]$	$2\cdot 10^{5}$
$\mathrm{Stiffness}\;\mathrm{of}\;\mathrm{rear}\;\mathrm{wheels},\;k_{2}\;\left[N/m\right]$	$2\cdot 10^{5}$
$\mathrm{Stiffness}\;\mathrm{of}\;\mathrm{front}\;\mathrm{suspension},\;k_{3}\;\left[N/m\right]$	2⋅24,236.5
$\mathrm{Stiffness}\;\mathrm{of}\;\mathrm{rear}\;\mathrm{suspension},\;k_{4}\;\left[N/m\right]$	2⋅16,965.5
$\mathrm{Damping}\;\mathrm{of}\;\mathrm{front}\;\mathrm{suspension},\;c_{3}\;\left[\mathrm{Ns}/m\right]$	2⋅2726.6
$\mathrm{Damping}\;\mathrm{of}\;\mathrm{rear}\;\mathrm{suspension},\;c_{4\;}\left[\mathrm{Ns}/m\right]$	2⋅1908.6
$\mathrm{Distance}\;\mathrm{from}\;\mathrm{front}\;\mathrm{wheel}\;\mathrm{to}\;\mathrm{the}\;\mathrm{centre}\;\mathrm{of}\;\mathrm{vehicle}\;\mathrm{body}\;\mathrm{mass},\;a_{1}\;\left[m\right]$	1.37
$\mathrm{Distance}\;\mathrm{from}\;\mathrm{the}\;\mathrm{rear}\;\mathrm{wheel}\;\mathrm{to}\;\mathrm{the}\;\mathrm{centre}\;\mathrm{of}\;\mathrm{vehicle}\;\mathrm{body}\;\mathrm{mass},\;a_{2}\;\left[m\right]$	1.33
Weight of centre of vehicle body mass	0.6
Wheel radii, r [m]	0.3
$\mathrm{Front}\;\mathrm{axle}\;\mathrm{moment}\;\mathrm{of}\;\mathrm{inertia},\;I_{f}\;\left[{\mathrm{kgm}}^{2}\right]$	2⋅1.155
$\mathrm{Rear}\;\mathrm{axle}\;\mathrm{moment}\;\mathrm{of}\;\mathrm{inertia},\;I_{r}\;\left[{\mathrm{kgm}}^{2}\right]$	2⋅0.77

**Table 2:** Confusion matrix for road pavement type and condition combinations.

	Predicted	C1	C2	C3	C4	C5	C6	Per Class Accuracy	Precision	Recall	F1 Score
Actual											
C1: Asphalt dry		174	14	4	4	2	2	87.0%	0.90	0.87	0.89
C2: Asphalt wet		3	191	0	4	0	2	95.5%	0.88	0.955	0.92
C3: Cobblestone dry		15	3	171	8	0	3	85.5%	0.96	0.855	0.90
C4: Cobblestone wet		1	9	1	188	0	1	94.0%	0.92	0.94	0.93
C5: Gravel dry		0	0	2	0	151	47	75.5%	0.90	0.755	0.82
C6: Gravel wet		0	0	0	0	15	185	92.5%	0.77	0.93	0.84

**Table 3:** Confusion matrix for road pavement type only.

	Predicted	Asphalt	Cobblestone	Gravel	Per Class Accuracy	Precision	Recall	F1 Score
Actual								
Asphalt		382	12	6	95.5%	0.93	0.955	0.94
Cobblestone		28	368	4	92%	0.96	0.92	0.94
Gravel		0	2	398	99.5%	0.975	0.995	0.98

**Table 4:** Confusion matrix for road conditions only.

	Predicted	Dry	Wet	Per Class Accuracy	Precision	Recall	F1 Score
Actual							
Dry		519	81	86.5%	0.96	0.865	0.91
Wet		20	580	96.7%	0.88	0.965	0.92

**Table 5:** Stopping distance.

Pavement Type	Max Friction Coefficient	Stopping Distance				
		Without ABS		Conventional ABS	SYSTEM with Preview	
		m	Compared to Conv. ABS, %	m	m	Compared to Conv. ABS, %
Dry asphalt	0.15	21.37	−64	13.05	13.05	0
Wet asphalt	0.11	32.45	−33	24.36	20.05	18
Dry cobble.	0.32	26.78	−42	18.89	17.51	7
Wet cobble.	0.20	33.30	−18	28.22	27.78	2
Dry gravel	0.3	25.66	−14	22.53	21.83	3
Wet gravel	0.4	31.79	8	34.68	30.05	13

**Table 6:** Stopping distance with wrong classification.

	Set Surface	Dry Asphalt	Wet Asphalt	Dry Cobble.	Wet Cobble.	Dry Gravel	Wet Gravel
Actual Surface							
Dry asphalt		13.05	13.51	19.40	19.17	19.40	20.37
Wet asphalt		24.36	20.05	30.01	27.82	29.59	31.09
Dry cobble.		18.89	20.78	17.51	17.87	17.53	18.31
Wet cobble.		28.22	29.55	28.34	27.78	28.16	29.24
Dry gravel		22.53	23.74	21.84	22.02	21.83	21.99
Wet gravel		34.68	38.39	30.32	32.34	30.48	30.05

**Figure A1:**
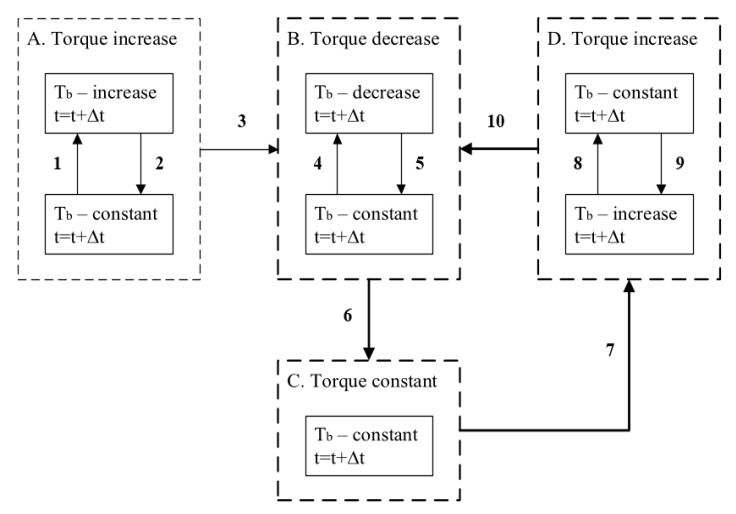
ABS algorithm.

## Appendix A

Total longitudinal force $F_{tot}$ of the vehicle with ABS can be calculated using Equation 7, taking into account that $\mu_{f}$ is controlled using the ABS algorithm presented in Figure A1 and Table A1. This algorithm was developed by the authors based on that created in TU Ilmenau and described in [39]. The operating principle of the ABS algorithm used is based on predefined wheel accelerations and slip thresholds. During the braking, the algorithm starts from stage A (Table A1) and regulates the braking torque based on wheel acceleration and its slip (Figure A1).

Since actual wheel slip becomes higher than the reference one, the algorithm switches to stage B. It should be noted that stage A is activated during initial braking only after that algorithm works in stages B, C, and D.

**Table A1:** Control strategy.

Stage	Rule	Stage	Rule
1	$\mathrm{if}\;a_{w}\;<\;\left[a_{min}\right]\;\&\;t\;>\;t_{up}$; t = 0	6	$\mathrm{if}\;a_{w}\;>\;\left[A\right]$; t = 0
2	$\mathrm{if}\;\lambda \;\;<\;\left[\;\lambda_{ref}\right]\;\&\;t\;>\;t_{up}$; t = 0	7	if $a_{w}\;>\;\left[a_{max}\right]\;\&\;\;\lambda \;<\;\left[\lambda_{ref}\right]$, $or\;t>t_{sw}$; t = 0
3	if $\lambda \;>\;\left[\;\lambda_{ref}\right]$; t = 0	8	if $\lambda \;<\;\left[\;\lambda_{ref}\right]\;\&\;t\;>\;t_{up};$ t = 0
4	if $a_{w}\;<\;\left[A\right]\;\&\;t\;>\;t_{down};$ t = 0	9	$\mathrm{if}\;\lambda \;<\;\left[\;\lambda_{ref}\right]\;\&\;t\;>\;t_{up}$; t = 0
5	if $a_{w}\;<\;\left[A\right]\;\&\;t\;>\;t_{sw}$; t = 0	10	$\mathrm{if}\;\lambda \;>\;\left[\;\lambda_{ref}\right]$; t = 0
